# Exposure to the Viral By-Product dsRNA or Coxsackievirus B5 Triggers Pancreatic Beta Cell Apoptosis via a Bim / Mcl-1 Imbalance

**DOI:** 10.1371/journal.ppat.1002267

**Published:** 2011-09-22

**Authors:** Maikel L. Colli, Tatiane C. Nogueira, Florent Allagnat, Daniel A. Cunha, Esteban N. Gurzov, Alessandra K. Cardozo, Merja Roivainen, Anne Op de beeck, Decio L. Eizirik

**Affiliations:** 1 Laboratory of Experimental Medicine, Medical Faculty, Université Libre de Bruxelles, Brussels, Belgium; 2 Intestinal Viruses Unit, Department of Infectious Disease Surveillance and Control, National Institute for Health and Welfare (THL), Helsinki, Finland; 3 Virology Unit, Medical Faculty, Université Libre de Bruxelles, Brussels, Belgium; Mount Sinai School of Medicine, United States of America

## Abstract

The rise in type 1 diabetes (T1D) incidence in recent decades is probably related to modifications in environmental factors. Viruses are among the putative environmental triggers of T1D. The mechanisms regulating beta cell responses to viruses, however, remain to be defined. We have presently clarified the signaling pathways leading to beta cell apoptosis following exposure to the viral mimetic double-stranded RNA (dsRNA) and a diabetogenic enterovirus (Coxsackievirus B5). Internal dsRNA induces cell death via the intrinsic mitochondrial pathway. In this process, activation of the dsRNA-dependent protein kinase (PKR) promotes eIF2α phosphorylation and protein synthesis inhibition, leading to downregulation of the antiapoptotic Bcl-2 protein myeloid cell leukemia sequence 1 (Mcl-1). Mcl-1 decrease results in the release of the BH3-only protein Bim, which activates the mitochondrial pathway of apoptosis. Indeed, Bim knockdown prevented both dsRNA- and Coxsackievirus B5-induced beta cell death, and counteracted the proapoptotic effects of Mcl-1 silencing. These observations indicate that the balance between Mcl-1 and Bim is a key factor regulating beta cell survival during diabetogenic viral infections.

## Author Summary

The global prevalence of type 1 diabetes (T1D) is approximately 20 million individuals, and projections indicate that this will double in the coming decades. This increase in T1D incidence is probably related to modification in the exposure to environmental factors. Viruses are one of the putative environmental agents that trigger T1D. The mechanisms leading to beta cell loss during viral infection, however, remain to be clarified. The present study aimed to clarify the mechanisms of dsRNA- and virus-induced beta cell demise. Thus, dsRNA produced during viral replication in beta cells induces activation of the kinase PKR. This kinase phosphorylates the elongation factor eIF2α promoting inhibition of protein synthesis which decreases the short-life antiapoptotic protein Mcl-1. Decline of Mcl-1 allows Bim to exert its proapoptotic effects by activation of the intrinsic pathway of apoptosis. In conclusion, we demonstrate that the balance Mcl-1/Bim is central for dsRNA- and enterovirus-induced beta cell apoptosis. This clarifies one of the key questions in early T1D pathogenesis, namely the mechanisms behind viral-induced beta cell apoptosis, and the subsequent triggering of insulitis.

## Introduction

Type 1 diabetes (T1D) is a chronic autoimmune disease characterized by the progressive and selective destruction of the insulin-producing pancreatic beta cells [1]. It mainly affects individuals during childhood or adolescence and requires a life-long treatment with insulin, which at the US represents a cost of $14.4 billion per year [2]. Triggering of diabetes results from an interaction between genetical and environmental factors. Most candidate genes for T1D are supposed to act at the immune system level [3], but we have recently shown that nearly 30% of these candidate genes are also expressed in beta cells and may modulate their responses after exposure to potential environmental factors [4]. These findings indicate that beta cells are not only targets, but also actors of T1D pathophysiology [4].

The incidence of T1D is increasing 3.9% per year in Europe, especially among the youngest population (<5 years-old) in which a doubling in the number of new cases is expected between 2005 and 2020 [5]. An explanation for this rapid augment in T1D incidence may be the increased exposure to diabetogenic environmental factors. Viruses are among the potential environmental causes of T1D [6], as suggested by epidemiological [7], experimental [8] and clinical data [9].

Enterovisuses (EV) such as Coxsackievirus B (CVB) [10], are the main candidates. Coxsackievirus B4 was isolated from a child who died of diabetic ketoacidosis and this virus induced diabetes in mice [11]. Among the Coxsackievirus, CVB5 is the most deleterious prototype strain for human pancreatic islets [12]. An increase of T1D incidence has been described after epidemics of CVB5 [13] and these epidemics are frequent in Finland, the country with the highest incidence of T1D [14].

The pathogenic role of viruses in T1D might involve damage to beta cells and the local induction of proinflammatory mediators [1]. CVB-infected pancreatic beta cells can be phagocyted by both macrophages [15] and dendritic cells [16], leading to activation of the immune system, presentation of islet autoantigens and release of cytokines/chemokines. Local injury of beta cells induced by dsRNA, a by-product of viral replication, promotes autoimmune diabetes in animal models [17]. Both the viral mimetic dsRNA [18] and Coxsackievirus [19] induce beta cell apoptosis, but the mechanisms involved remain to be clarified.

Apoptotic cell death can be initiated by two signaling cascades, namely the intrinsic and the extrinsic pathways. The intrinsic pathway is the main pathway for endoplasmic reticulum (ER) stress and cytokine-induced beta cell apoptosis [20], [21], [22], [23]. In this pathway, a disequilibrium between the antiapoptotic and the proapoptotic Bcl-2 family members [24] leads to activation of Bcl-2-associated X protein (BAX) and Bcl-2 antagonist or killer (BAK) and the consequent permeabilization of mitochondrial outer membrane. This allows cytochrome *c* release to the cytoplasm and the assembly of the apoptosome. The apoptosome then recruits and activates the initiator caspase 9, which cleaves and activates the effector caspase 3, leading to execution of apoptosis [25].

We have presently investigated the molecular pathways involved in dsRNA and viral-induced beta cell apoptosis. Similar to ER stress and cytokine-induced apoptosis [24], the viral by-product dsRNA activates the intrinsic pathway of apoptosis. The nature of the Bcl-2 family members involved is, however, different. Thus, dsRNA-dependent protein kinase (PKR) activation by cytoplasmic dsRNA leads to phosphorylation of the elongation factor eIF2α. The eIF2α phosphorylation promotes inhibition of protein translation, which is followed by an early and progressive decrease in the expression of the antiapoptotic myeloid cell leukemia sequence 1 (Mcl-1) protein. Decreased expression of Mcl-1 allows the proapoptotic protein Bim to activate BAX, leading to cytochrome *c* release, caspase 9 and 3 activation and beta cell apoptosis. Similar findings were observed during viral infection of pancreatic beta cells by CVB5. These observations clarify the mechanisms involved in viral-induced apoptosis in pancreatic beta cells, and suggest that usage of Bcl-2 proteins is context-dependent during beta cell apoptosis initiated by different stimuli.

## Results

### Internal dsRNA induces beta cell apoptosis via the intrinsic mitochondrial pathway of apoptosis

**Figure 1:**
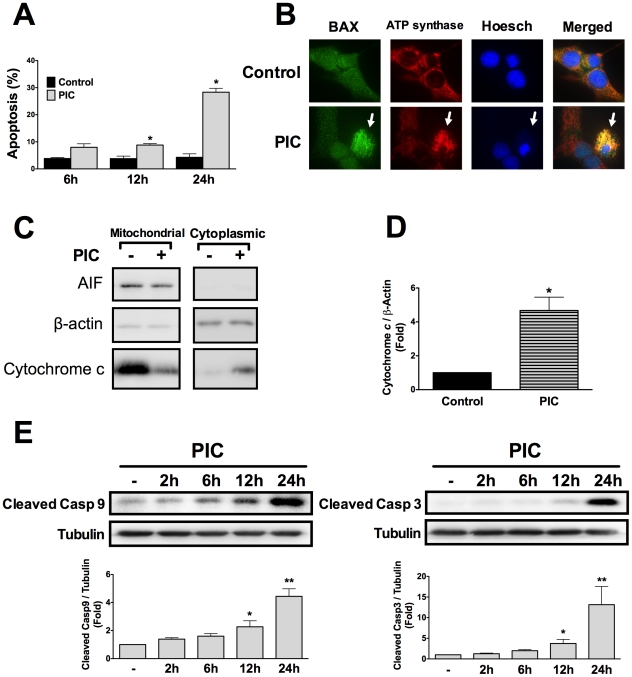
Internal dsRNA induces beta cell death via the mitochondrial pathway of apoptosis. ***A.*** INS-1E cells were transfected with the synthetic dsRNA polyinosinic-polycytidylic acid (PIC) and apoptosis was evaluated at different time points using nuclear dyes as described in Methods (n = 5, *P<0.01 vs. untreated). ***B.*** BAX localization was studied by immunocytochemistry in INS-1E cells exposed or not to internal dsRNA for 24h. Pictures are representative of 4 independent experiments; arrows indicate BAX mitochondrial localization, similar to the ATP Synthase (mitochondrial marker) ***C.*** Cytochrome *c* release in INS-1E cells treated for 24h in the presence or absence of intracellular dsRNA. Pictures are representative of 4 independent experiments. ***D.*** Western blot quantified by densitometry of cytoplasmic protein fractions in INS-1E cells transfected or not with dsRNA for 24h (n = 4, *P<0.01 vs. untreated). ***E*** **.** Western blot analysis and densitometry of cleaved caspases 9 and 3 after INS-1E cells exposure to internal dsRNA at different time points (n = 5–6, *P<0.05, **P<0.01 vs. untreated). Data are mean ± SEM.

Transfection with the synthetic dsRNA polyinosinic-polycytidylic acid (PIC) induced beta cell apoptosis already after 12h, with progressive increase up to 24h (Figure 1A). To characterize the pathways involved in beta cell apoptosis, BAX translocation to the mitochondria was analyzed by immunocytochemistry. Intracellular dsRNA promoted the translocation of BAX (Figure 1B) and the release of cytochrome *c* from the mitochondria to the cytoplasm (Figures 1C and D). This activated the initiator caspase 9 and effector caspase 3 (Figure 1E), characterizing induction of the intrinsic mitochondrial pathway of apoptosis.

### Expression of antiapoptotic Bcl-2 family members regulates beta cell apoptosis and is decreased upon exposure to internal dsRNA

**Figure 2:**
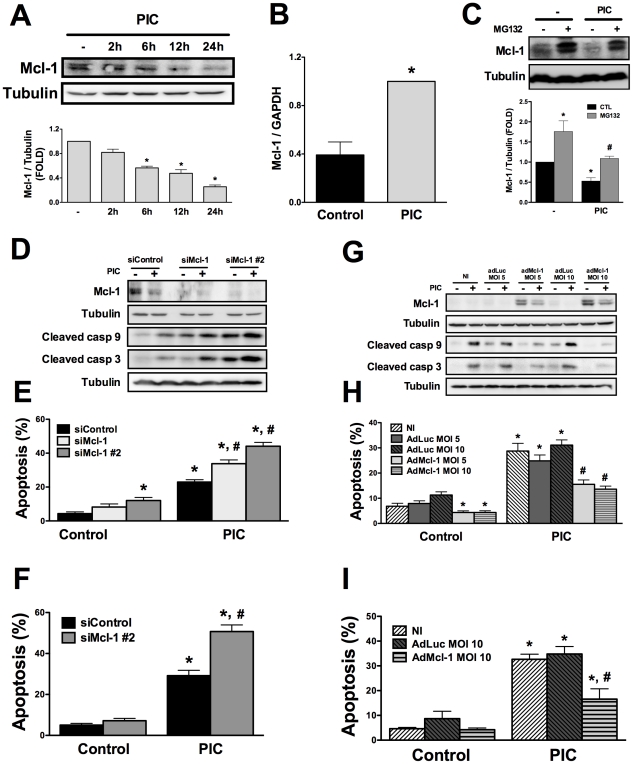
Mcl-1 protein expression is decreased by internal dsRNA and its modulation regulates beta cell apoptosis. ***A.*** INS-1E cells were transfected with dsRNA for different time points and Mcl-1 expression was analyzed by Western blot (top) and quantified by densitometry (bottom; data are shown as fold-change compared to non-treated cells; -) (n = 3, *P<0.01 vs. untreated). ***B.*** mRNA expression of Mcl-1 in INS-1E cells treated or not with internal dsRNA for 24h (n = 5, *P<0.01 vs. untreated). Results are corrected for the housekeeping gene GAPDH. ***C.*** INS-1E cells were treated or not for 12h with the proteasome inhibitor MG-132 in the presence or absence of intracellular PIC and Mcl-1 expression was analyzed by Western blot (top) and quantified by densitometry (bottom; data are shown as fold-change compared to non-treated cells; -) (n = 4, *P<0.01 vs. control (untreated), #P<0.05 vs. PIC alone). ***D, E*** *and* ***F.*** INS-1E cells (D and E) or FACS-purified primary beta cells (F) were transfected with siControl or with two different small-interfering RNAs against Mcl-1 (siMcl-1 and siMcl-1 #2) for 48h, as described in Methods. ***D.*** Protein expression of Mcl-1 and cleaved caspases 9 and 3 was evaluated by Western blot. Pictures are representative of 3 independent experiments. ***E*** *and* ***F.*** dsRNA-induced apoptosis in INS-1E cells (E) and FACS-purified primary beta cells (F) treated for 24 h with PIC with or without Mcl-1 knockdown (n = 5, *P<0.01 vs. siControl, #P<0.01 vs. siControl + PIC). ***G, H*** *and* ***I.*** INS-1E cells (G and H) or FACS-purified primary beta cells (I) were infected or not (NI) with adenovirus encoding the luciferase gene (adLuc) or rat Mcl-1 (adMcl-1) at different multiplicities of infection (MOI) for 48h, as indicated. ***G.*** Protein expression of Mcl-1 or cleaved caspases 9 and 3 were evaluated by Western blot 24h after PIC exposure. Pictures are representative of 3 independent experiments. ***H*** *and* ***I.*** dsRNA-induced apoptosis in INS-1E cells (H) and FACS-purified primary beta cells (I) treated for 24h with PIC with or without Mcl-1 overexpression (n = 4, *P<0.01 vs. NI, #P<0.01 vs. NI + PIC). Data are mean ± SEM.

The antiapoptotic Bcl-2 protein Mcl-1 is an important regulator of beta cell death after exposure to proinflammatory cytokines and ER stressors [20]. Mcl-1 protein expression gradually decreased from 6h until 24h after PIC transfection (Figure 2A). This reduction in Mcl-1 protein was a post-transcriptional effect since Mcl-1 mRNA expression was in fact increased at 24h (Figure 2B). Mcl-1 is targeted for degradation at the proteasome [26]. INS-1E cells treated with the proteasome inhibitor MG-132 (same experimental conditions as in [20], [22]) had increased basal Mcl-1 expression, and the inhibitor partially prevented dsRNA-induced decrease in Mcl-1 expression (Figure 2C), suggesting that proteasomal degradation contributes at least in part for the observed Mcl-1 downregulation. Mcl-1 knockdown by two previously validated siRNAs [20] exacerbated cleavage of caspases 9/3 (Figure 2D) and PIC-induced apoptosis in INS-1E cells (Figure 2E) and FACS-purified primary beta cells (Figure 2F). On the other hand, overexpressing rat Mcl-1 by the use of an adenoviral vector (adMcl-1 [20]) prevented the activation of caspases 9/3 and reduced by > 40% apoptosis induced by dsRNA (Figures 2G, H and I). This is a specific effect, since infection with a control adenovirus encoding luciferase (adLuc) did not modify apoptosis (Figures 2H and I) and Mcl-1 silencing or overexpression did not modify expression of Bcl-XL or Bcl-2 (data not shown). These observations indicate a key role for Mcl-1 decrease in dsRNA-induced apoptosis.

The two other key antiapoptotic Bcl-2 proteins in beta cells are Bcl-XL and Bcl-2 [24]. Bcl-XL but not Bcl-2 protein expression was decreased after PIC exposure (Supplemental Figures S1A and B). This downregulation, however, was only detected at later time-points (after 12h and 24h) and to a lesser extent than observed with Mcl-1 (compare Figure 2A and Supplemental Figure S1A). Bcl-XL and Bcl-2 knockdown (Supplemental Figures S1C and D) increased basal beta cell apoptosis from 7% to more than 30% and further increased PIC-induced apoptosis, as compared to siControl (Supplemental Figures S1E and F). As observed with siMcl-1, inhibition of Bcl-XL and Bcl-2 promoted caspases 9 and 3 cleavage in parallel with beta cell apoptosis (Supplemental Figures S1G and H). Similar results were observed with second independent siRNAs against Bcl-XL and Bcl-2 (Supplemental Figure S2). Nevertheless, when the effects of Bcl-XL or Bcl-2 silencing on PIC-induced apoptosis were corrected by their basal levels of apoptosis using the apoptotic index (i.e. siBcl-XL or Bcl-2 + PIC correct by siBcl-XL or Bcl-2 alone [27]) they were not different from the control group (siControl + PIC) (Supplemental Figure S3). This finding suggests that knocking down these proteins has only additive effect to PIC-induced apoptosis. On the other hand, Mcl-1 knockdown using two different siRNAs resulted in increased apoptotic indexes as compared to siControl, indicating that silencing of Mcl-1, different from Bcl-XL and Bcl-2, potentiates apoptosis caused by PIC (Supplemental Figure S3A).

### The proapoptotic BH3-only protein Bim regulates pancreatic beta cell apoptosis after dsRNA exposure

**Figure 3:**
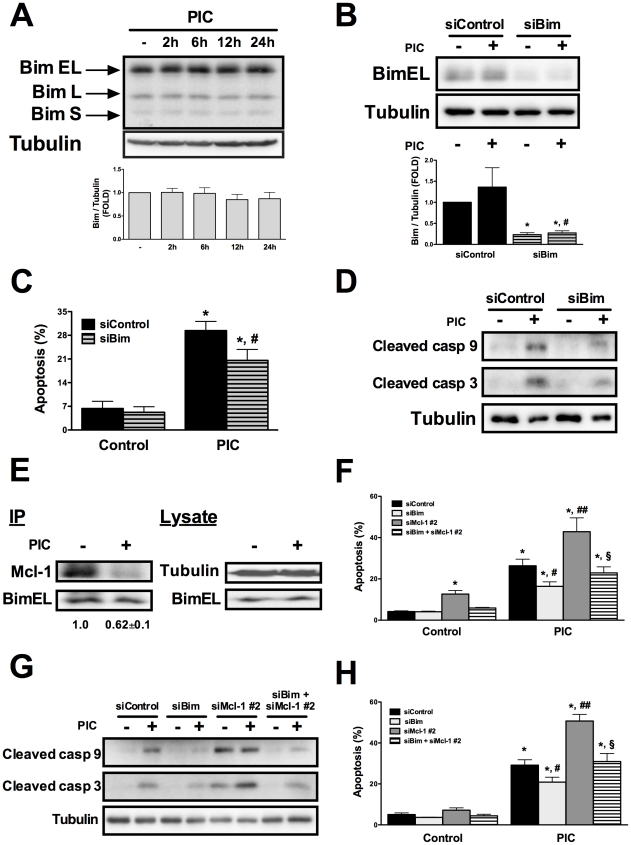
Bim contributes for dsRNA-induced apoptosis and its silencing prevents the proapoptotic effects of Mcl-1 knockdown. ***A.*** Time course analysis of Bim protein expression in INS-1E cells treated with internal dsRNA. Cells were harvested at different time points and Bim expression was evaluated by Western blot (top) and quantified by densitometry (bottom) (n = 10). ***B*** *to* ***D.*** INS-1E cells were transfected with siControl or a specific siRNA against Bim (siBim) for 24 h and then exposed to intracellular dsRNA for 24h. Bim knockdown was confirmed by Western blot (B). Cell viability (C) was measured using nuclear dyes (n = 3, *P<0.01 vs. siControl, #P<0.05 vs. siControl + PIC). Activation of caspases 9 and 3 (D) was determined in the presence or not of Bim silencing. ***E.*** INS-1E cells were left untreated or transfected with dsRNA for 24h and then subjected to immunoprecipitation with an antibody against Bim (IP). The presence of Mcl-1 and Bim in the immunoprecipitated material was determined using specific antibodies, and the ratio Mcl-1/Bim band intensity determined by densitometry. The total lysate (Lysate) was evaluated by Western blotting with anti-Bim and anti-α-tubulin antibodies. The pictures shown are representative of 3–4 independent experiments. INS-1E cells (F and G) or FACS-purified primary beta cells (H) were transfected with siControl, siBim, siMcl-1 or a combination of siBim plus siMcl-1. After a 24h recovery period they were then exposed to internal dsRNA for 24 h. Cell viability (F and H) and cleavage of caspases 9 and 3 (G) were determined after 24h exposure to dsRNA (n = 3 - 5, *P<0.01 vs. siControl, #P<0.05 vs. siControl + PIC, ##P<0.01 vs. siControl + PIC, §P<0.01 vs. siMcl-1 + PIC). The pictures shown are representative of 3 independent experiments. Data are mean ± SEM.

The BH3-only protein Bim was previously shown to be an important regulator of glucose-mediated beta cell apoptosis [28] and virus-induced cell death in other cell types [29]. Against this background, we evaluated its role in dsRNA-induced beta cell apoptosis. Time-course experiments with INS-1E cells exposed to intracellular PIC did not show modifications in the expression of the Bim isoforms EL, L and S (Figure 3A). Since Bim may modulate apoptosis independently of changes in its expression [30], we next evaluated whether Bim knockdown affects dsRNA-induced apoptosis. The use of a specific siRNA against Bim inhibited by > 70% its protein expression at both basal and PIC-induced conditions (Figure 3B). Bim knockdown prevented PIC-induced apoptosis in INS-1E cells (Figure 3C) and in primary beta cells (Figure 3H). These results were confirmed with a second siRNA against Bim (Supplemental Figure S4). In agreement with the effects on cell viability, PIC-induced activation of caspases 9 and 3 was also reduced after inhibition of Bim (Figure 3D). We studied two other proapoptotic BH3-only proteins, namely Death Protein-5 (DP5) and p53 Up-regulated Modulator of Apoptosis (PUMA), previously shown to regulate cytokine-induced beta cell apoptosis [21], [31]. We obtained a knockdown of >80% for DP5 and >75% for PUMA mRNA expression (Supplemental Figures S5A and C); however, differently from what was observed following Bim silencing, neither siDP5 nor siPUMA prevented PIC-triggered apoptosis (Supplemental Figures S5B and D).

One of the mechanisms by which the antiapoptotic Bcl-2 proteins act is via binding and inactivating specific proapoptotic BH3-only proteins [32]. We thus performed co-immunoprecipitation studies to evaluate the association between Bim and Mcl-1. Bim was immunoprecipitated using a specific polyclonal antibody and the presence of Mcl-1 in the precipitated material was determined with an anti-Mcl-1 antibody. We observed that at basal condition Bim was bound to Mcl-1, but after 24h of PIC exposure there was a clear decrease in the association between these two proteins, confirming that Bim is liberated from Mcl-1 following dsRNA exposure (Figure 3E).

To evaluate whether the PIC-induced decrease in Mcl-1 protein expression contributes to beta cell apoptosis by hampering Bim inactivation by Mcl-1, we performed double-knockdown (siMcl-1 + siBim) and evaluated apoptosis and cleavage of caspases 9 and 3 in comparison to single knockdown (siMcl-1 or siBim). The combined use of siMcl-1 plus siBim produced a >70% suppression of the target genes, as observed with the use of these siRNAs individually (data not shown). Interestingly, the double-silencing of siMcl-1 + siBim prevented the proapoptotic effect caused by Mcl-1 knockdown in INS-1E cells (Figures 3F and G) and primary beta cells (Figure 3H), suggesting that the release of Bim from the Mcl-1/Bim complex induced by both dsRNA and siMcl-1 is a key effector of beta cell apoptosis.

### Infection of beta cells by Coxsackievirus B5 induces the mitochondrial apoptosis pathway through the BH3-only protein Bim

**Figure 4:**
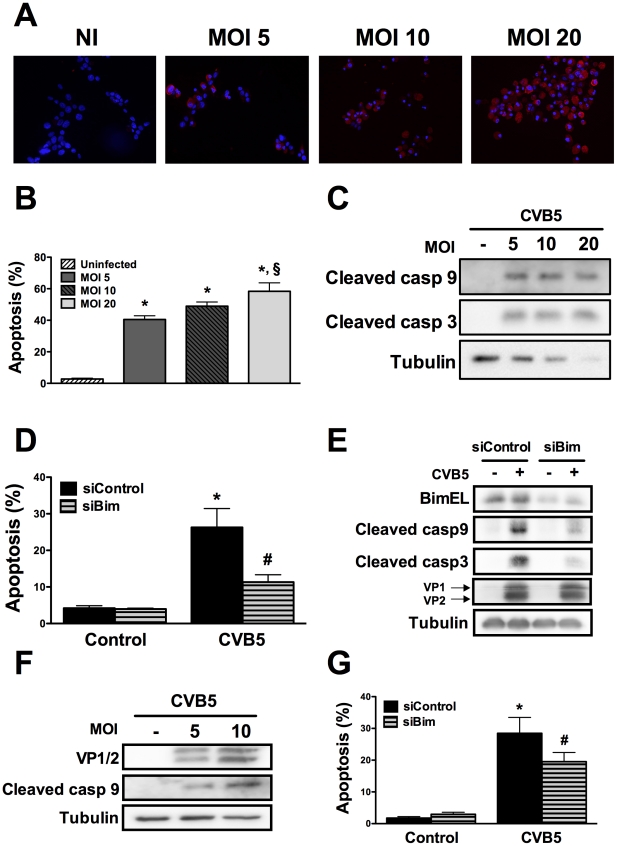
CVB5 infection induces apoptosis in beta cells via the BH3-only protein Bim. ***A*** *to* ***G.*** INS-1E cells (A to E) or FACS-purified primary beta cells (F and G) were infected with CVB5 at the indicated MOIs, as described in Methods. Protein expression of the viral capsids was evaluated by immunocytochemistry at 24h using an enterovirus-specific antibody (A). Cell apoptosis (B) and caspases 9 and 3 cleavage (C) were determined in cells infected or not with CVB5 for 24 h. (n = 3, *P<0.01 vs. uninfected, §P<0.01 vs. MOI 5). ***D*** *to* ***G.*** INS-1E cells (D) or primary beta cells (G) were transfected with siControl or a specific siRNA against Bim (siBim) for 24 h and then infected with CVB5 at the MOI 5. Cell viability was determined after 18h (D and G) (n = 3, *P<0.01 vs. siControl, #P<0.05 vs. siControl + PIC). Bim knockdown, caspases 9 and 3 cleavage and the expression of the viral capsids (VP1 and 2) were evaluated by Western blot (E and F). The pictures shown are representative of 3 independent experiments. Data are mean ± SEM.

To assess whether the above described pathways are also relevant in the context of a viral infection of beta cells, we infected INS-1E cells and FACS-purified rat beta cells with Coxsackievirus B5. First, we confirmed that this enterovirus infects rat beta cells at the different MOIs tested by determining the presence of the viral capsid (VP1 and 2) (Figures 4A, E and F). The viral capsid was already detected in the cells at 8h post-infection and its expression increased further up to 24h (data not shown; Figures 4A and F). Infection of beta cells at these MOIs induced cell death mainly by apoptosis (Figure 4B) via the intrinsic pathway (Figures 4C and F), as observed with internal dsRNA (Figure 1). In line with these observations, Bim silencing significantly decreased virus-induces apoptosis (Figures 4D and G) and caspase 9 and 3 activation (Figure 4E). Importantly, inhibition of virus-induced apoptosis by knocking down Bim did not modify the amount of viral capsids (VP1 and 2; Figure 4E), indicating that, under the present experimental conditions, inhibition of apoptosis did not exacerbate viral replication in beta cells. These observations suggest that both dsRNA and the potentially diabetogenic virus CVB5 induce a similar pathway of apoptosis in beta cells. Interestingly, both dsRNA and CVB5 induced expression of interferon β (Supplemental Figure S6A and B) and interferon α (data not shown), but with different kinetics. Expression of interferon β mRNA was several-fold higher in primary beta cells as compared to INS-1E cells (Supplemental Figure S6C). This increased expression of type I interferons may also contribute to the observed *in vitro* beta cell apoptosis, as suggested by previous studies [33].

### dsRNA-induced decrease in Mcl-1 protein expression depends on eIF2α phosphorylation by dsRNA-dependent protein kinase (PKR)

**Figure 5:**
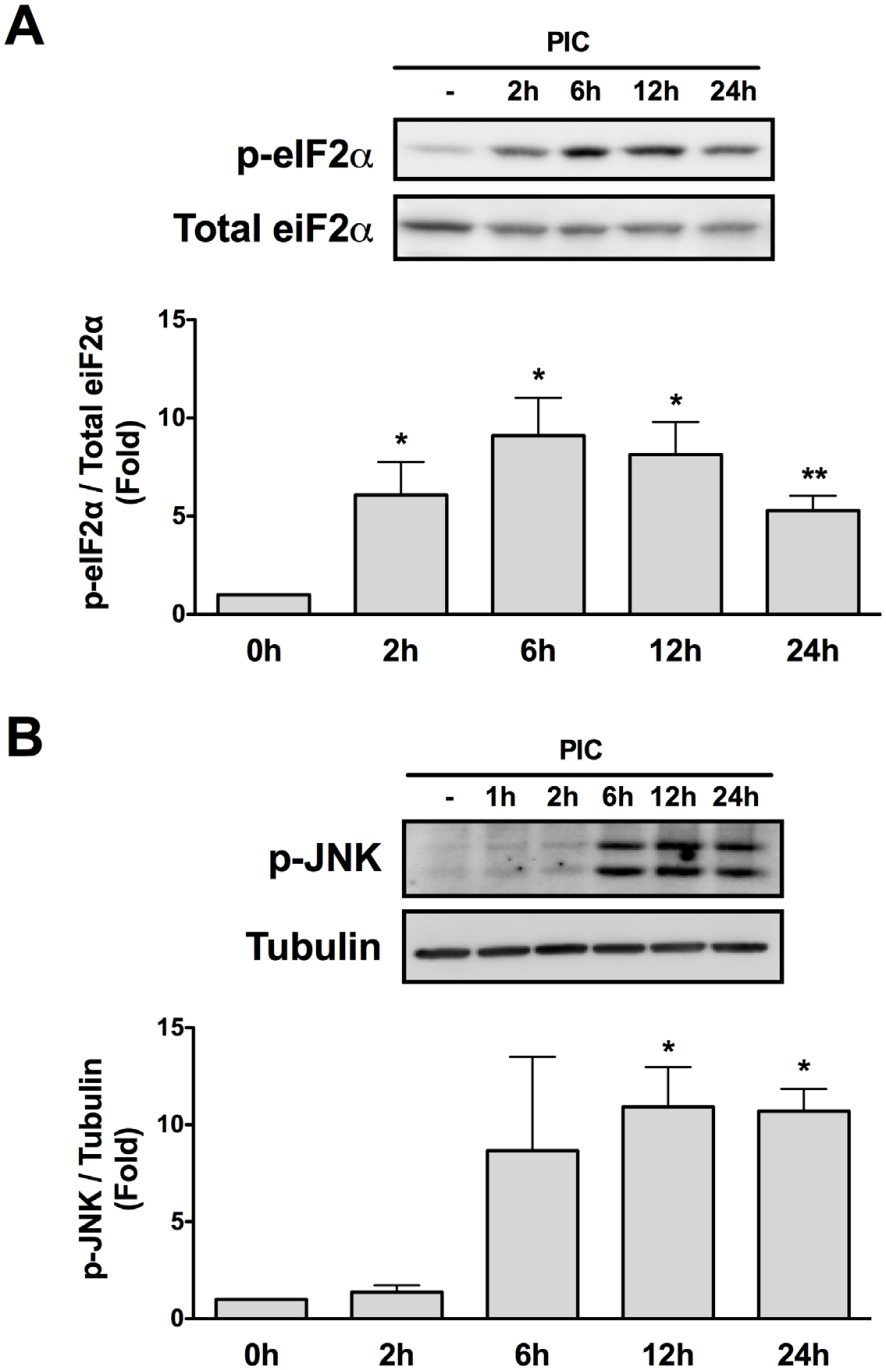
Internal dsRNA induces eIF2α and JNK phosphorylation. Cell lysates of INS-1E cells transfected with dsRNA for different time points were used for Western blotting with antibodies against p-eIF2α (A) or p-JNK (B). Densitometry was performed and the results were normalized for the expression of total eIF2α (A) or α-tubulin (B) (n = 5, *P<0.01 vs. untreated, **P<0.05 vs. untreated). The pictures shown are representative of 5 independent experiments. Data are mean ± SEM.

Two important regulators of Mcl-1 protein expression have been reported in pancreatic beta cells, namely phosphorylation of eIF2α and c-Jun N-terminal Kinase (JNK) activation [20]. Taking this into account we evaluated the time course phosphorylation of eIF2α and JNK in INS-1E cells transfected with PIC. Both eIF2α and JNK were activated by PIC, but with different profiles. Thus, eIF2α phosphorylation started earlier (2h), had a peak at 6h then slowly decreased up to 24h (Figure 5A); JNK phosphorylation started later (6h), reached a plateau at 12h and remained upregulated up to 24h (Figure 5B).

JNK inhibition by a specific chemical inhibitor [34], did not prevent Mcl-1 protein decrease (Supplemental Figure S7). We also evaluated the expression of the BH3-only protein Noxa previously shown to induce proteosomal degradation of Mcl-1 [35], but did not detect its expression in beta cells (data not shown).

**Figure 6:**
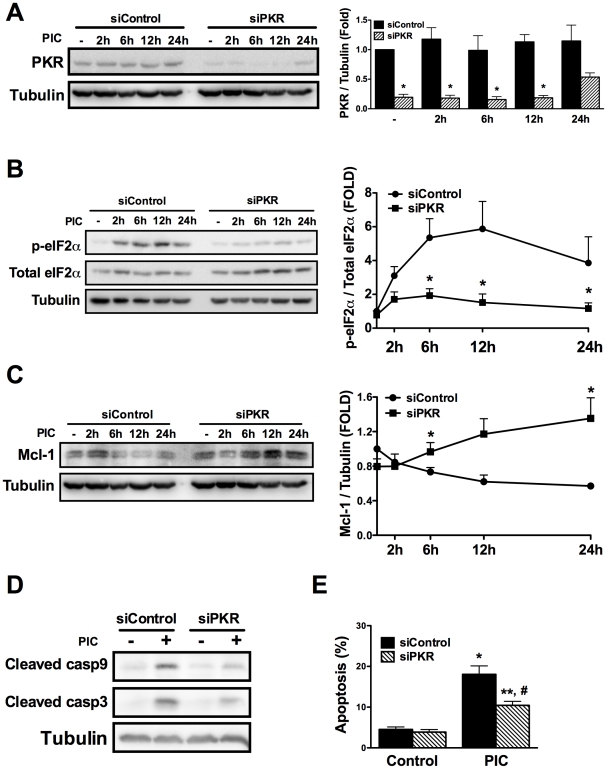
dsRNA-induced decrease in Mcl-1 protein expression depends on eIF2α phosphorylation by PKR. ***A*** *to* ***E.*** Cells were transfected with siControl or a siRNA against PKR (siPKR) and 48h after recovery treated with internal dsRNA for different time points. The expression of PKR (A), p-eIF2α (B) and Mcl-1 (C) was determined by Western blot (left) and quantified by densitometry (right) in INS-1 E cells (n = 4, *P<0.01 vs. siControl + PIC at the same time point). Cleavage of caspase 9 and 3 (D) and cell viability (E) were evaluated after 24h of dsRNA exposure with or without siPKR (n = 5, *P<0.01 vs. siControl, **P<0.05 vs. siControl, #P<0.01 vs. siControl + PIC). The pictures shown are representative of 4-5 independent experiments. Data are mean ± SEM.

PKR-like endoplasmic reticulum kinase (PERK) and PKR were evaluated as two possible mediators of the eIF2α phosphorylation induced by PIC [36], [37]. PERK is a kinase activated by endoplasmic reticulum (ER) stress [36], while PKR is a classic downstream response to dsRNA/viruses [37]. Thapsigargin, a well-know ER stressor [34], was used as positive control for PERK activation. Differently from thapsigargin, PIC did not induce PERK phosphorylation, while it clearly induced eIF2α phosphorylation (Supplemental Figures S8A and B), indicating that another upstream kinase is responsible for this effect. We thus evaluated the role of PKR in this process by using a specific siRNA against PKR which inhibited PKR protein expression by >80% (Figure 6A). Knockdown of PKR almost completely prevented PIC-induced eIF2α phosphorylation and the dsRNA-induced Mcl-1 decrease (Figures 6B and C); there was even an increase in Mcl-1 expression under this condition. In line with the observed increase in Mcl-1 protein expression, silencing of PKR reduced caspases 9 and 3 cleavage (Figure 6D) and PIC-induced apoptosis (Figure 6E). Similar results were obtained with a second siRNA against PKR (data not shown), confirming the key role for PKR in dsRNA-induced Mcl-1 decrease and consequent beta cell apoptosis.

## Discussion

**Figure 7:**
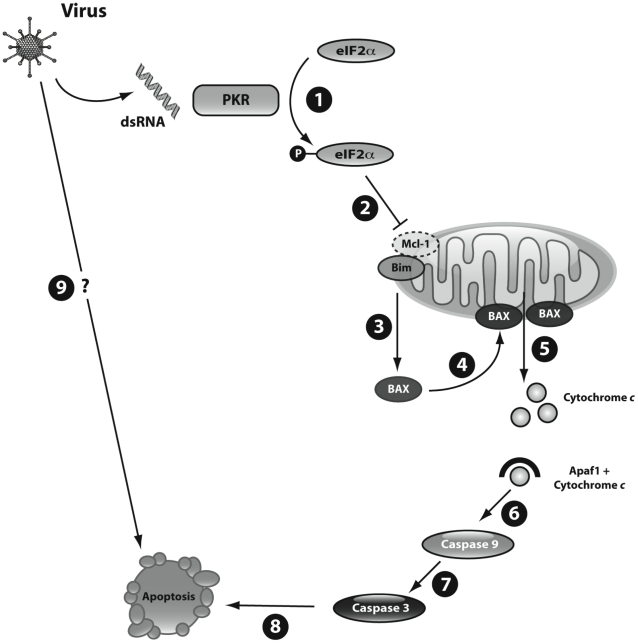
Schematic representation of dsRNA- and virus-induced beta cell apoptosis. During viral infection of beta cells the viral by-product dsRNA is released into the cytoplasm. **1**) Cytoplasmic dsRNA activates the kinase PKR that in turn phosphorylates the elongation factor eIF2α; **2**) phosphorylation of eIF2α leads to inhibition of protein translation with consequent decrease of Mcl-1 expression; **3**) reduction of Mcl-1 protein increases unbound Bim, which activates BAX; **4**) BAX translocates to the mitochondria; **5**) BAX oligomerizes and induces permeabilization of the mitochondrial outer membrane, allowing the release of cytochrome *c* to the cytoplasm; **6**) cytoplasmic cytochrome *c* forms complexes with Apaf1 and activates the initiator caspase 9; **7**) finally, caspase 9 cleaves and activates the executioner caspase 3, **8**) triggering the final steps of beta cell apoptosis; **9**) further studies are required to clarify additional pathways implicated in virus-induced beta cell apoptosis.

We presently show that intracellular dsRNA activates the intrinsic pathway of apoptosis in pancreatic beta cells. Triggering of apoptosis by dsRNA is secondary to an early and sustained downregulation of Mcl-1 protein expression (Figure 7), resulting in the release of the proapoptotic protein Bim. The unbound Bim then activates BAX translocation to the mitochondria, mitochondrial permeabilization, cytochrome *c* release, caspases 9 and 3 activation and beta cell apoptosis. Key results obtained with dsRNA, an intermediary product of viral replication, where partially confirmed in the context of a beta cell infection by the diabetogenic enterovirus CVB5.

Apoptosis is the main form of cell death in T1D [38]. Several virus-induced human diseases are associated with increased apoptosis in the target cells, including HIV1-associated dementia [39], cytomegalovirus encephalitis [40] and viral myocarditis [41]. Coxsakieviruses can induce beta cell death by different mechanisms, depending on the strain and multiplicity of infection (MOI) used [19]. At higher MOIs (>100) necrosis is the preferential mechanism of cell death, but at lower MOIs a shift towards apoptosis is observed [19]. Viral triggering of apoptosis depends on both the host and the virus [42]. The host regulates viral-induced apoptosis trough the local production of pro-inflammatory cytokines and chemokines [42]. Viral factors leading to apoptosis include dsRNA [43]. This viral nucleic acid is recognized by receptors such as the toll-like receptor 3 (TLR3), the kinase PKR, the helicases melanoma differentiation-associated gene 5 (MDA5), (which is also a candidate gene for T1D [44]) and retinoic-acid-inducible protein 1 (RIG-I); these receptors activate genes involved in both antiviral responses and apoptosis [45]. All these receptors are expressed in pancreatic beta cells [4], [18]. The pathways downstream of viral recognition/signaling leading to beta cell apoptosis, however, remained to be clarified. The present observations provide a coherent hypothesis for the mechanisms triggering beta cell apoptosis following a viral infection (Figure 7).

Mcl-1 is a pro-survival Bcl-2 family member with a short half-life (30–180 min), which makes it specially susceptible to changes in protein translation [35]. Mcl-1 is degraded in the proteasome [26], and use of a proteasome inhibitor prevented, at least in part, dsRNA-induced decrease in Mcl-1 expression (present findings). We also observed that intracellular dsRNA induces an early and sustained phosphorylation of eIF2α which, as previously shown by our group, leads to a decrease in total protein translation [18]. This eIF2α phosphorylation is mediated by PKR, a protein kinase mainly activated by dsRNAs produced during viral replication [37], but not by PERK (present data). Protein translation inhibition contributes to an early and progressive decrease in Mcl-1 protein levels, which is reverted by knocking down PKR. PKR silencing actually increases Mcl-1 protein expression, probably due to the “release” of protein translation combined with an increase in Mcl-1 mRNA induced by dsRNA (Figure 2B). PKR silencing also prevents dsRNA-induced apoptosis, at least partially due to this increase in Mcl-1 protein. Mcl-1 protein stability is partly regulated by multiple sites of phosphorylation, and JNK-mediated phosphorylation of Mcl-1 increases the rate of protein degradation [35]. We recently described that JNK activation contributes to Mcl-1 degradation in beta cells exposed to the cytokines interleukin-1β + interferon-γ [20]. Here, we observed that dsRNA induces JNK phosphorylation in beta cells, but JNK inhibition does not prevent Mcl-1 degradation. These findings indicate that dsRNA mainly regulates Mcl-1 protein expression via inhibition of protein translation.

Mcl-1 functions as a prosurvivor factor by neutralizing specific propapoptotic BH3-only proteins; Bim has been described as a preferential target of Mcl-1 in other cell types [32]. Bim is a BH3-only protein that presents features of both a “sensitizer”, i.e. it binds to antiapoptotic Bcl-2 protein and displaces the effectors BAX and BAK [32], and also as an “activator”; i.e. through directly binding to BAX and BAK it promotes their activation and the induction of the mitochondrial apoptosis pathway [46]. An imbalance between Mcl-1 and Bim has been shown to trigger apoptosis in other cell types in the context of cytokine deprivation [47] and granzyme B [48] activation. We presently observed that Bim is neutralized by Mcl-1 under basal condition. After dsRNA exposure or the use of specific siRNAs against Mcl-1, however, there is an increase in “free” Bim that subsequently activates BAX and triggers apoptosis (Figure 7). This is suggested by the observation that a double knockdown of Bim plus Mcl-1 (Figure 3) reverts the proapoptotic effects of Mcl-1 silencing. Bim can also mediate the pro-apoptotic effects of type I interferons produced during viral infections [49] and it is targeted by viruses to evade apoptosis in the host cells [50]. Type I interferons were induced during both dsRNA and CVB5 exposure (Supplemental Figure S6). Importantly, inhibition of Bim by specific siRNAs prevented caspases 9/3 activation and apoptosis in beta cells infected with the diabetogenic virus CVB5, confirming the key role of Bim during viral infection of beta cells.

Apoptosis is one of the mechanisms by which the host eliminates virus-infected cells and blocks viral spread [51]. In postmitotic and poorly proliferating cells such as neurons and beta cells, apoptosis might also promote functional loss and disease. Indeed, studies in viral-induced neuronal disease demonstrate that inhibition of apoptosis may reduce disease severity without changes in virus titers [52], [53]. In line with these findings, apoptosis prevention in beta cells by knocking down Bim did not modify viral replication *in vitro*.

In conclusion, we presently show that decreased expression of the anti-apoptotic protein Mcl-1, coupled to activation of the pro-apoptotic protein Bim, contributes to beta cell apoptosis during *in vitro* exposure to the viral mimetic dsRNA. In case these observations can be confirmed in future *in vivo* experiments, novel strategies to increase Mcl-1 or decrease Bim expression in pancreatic beta cells [24] may turn to be interesting approaches to protect beta cells during infection by putative “diabetogenic” viruses.

## Methods

### Ethics statement

This study was carried out in strict accordance with the recommendations in the Belgian Regulations for Animal Care guidelines. The protocol was approved by the Commission d'Ethique du Bien-Être Animal (CEBEA) on the Ethics of Animal Experiments of the Université Libre de Bruxelles (Permit Number: LA 1230351). All procedures was performed under Isoflurane anesthesia, and all efforts were made to minimize suffering.

### Culture of INS-1E cells and primary rat beta cells

The rat pancreatic beta cell line INS-1E (passages 57–75; a kind gift from Dr. C. Wollheim, Centre Medical Universitaire, Geneva, Switzerland) was cultured in medium containing RPMI 1640 GlutaMAX-I and 5% heat-inactivated fetal bovine serum (FBS) [54].

Male Wistar rats (Charles River Laboratories, Brussels, Belgium) were housed and used according to the Belgian Regulations for Animal Care guidelines. Rat islets were isolated by collagenase digestion of the pancreases. In order to obtain purified beta cells, the islets were dispersed and submitted to autofluorescence-activated cell sorting (FACS Aria, BD Bioscience, San Jose, USA) as previously described [33], [55]. The preparations used in the present study contained 93±2% of beta cells (n = 10). FACS-purified beta cells were cultured in Ham's F-10 medium containing 10 mM glucose, 5% FBS, 0.5% charcoal-absorbed bovine serum albumin (BSA Fraction V, Boehringer, Indianapolis, USA) [33]. During dsRNA and siRNAs transfection cells were cultured in medium without antibiotics and BSA.

### RNA interference

The sequences of the siRNAs used are provide in Supplemental Table S1. As control we used Allstars Negative Control siRNA (Qiagen, Venlo, Netherlands). We have previously shown that this negative control siRNA does not affect beta cell gene expression or insulin release as compared to non-transfected cells [20], [56]. The optimal conditions for siRNAs transfection were determined by using a FITC-coupled siRNA (Thermo Scientific) and functional/viability tests [57]. Cells were transfected overnight with 30 nM of siRNAs using Lipofectamine 2000 or RNAiMAX (Invitrogen, CA, USA) according to the manufacturer's instructions [56]. After a recovery period of 24 to 48 h, cells were treated with a synthetic dsRNA or infected by an enterovirus.

### Double-stranded RNA transfection, JNK and proteasome inhibition

The synthetic dsRNA polyinosinic-polycytidylic acid (PIC; Sigma, St. Louis, USA) was used at the concentration of 1 µg/ml [18]. All experiments were made with intracellular PIC, achieved via cell transfection under the same conditions as for siRNAs. Cells exposed to the transfectant alone were used as Control. Since cytoplasmic helicases can recognize dsRNA molecules based on their length [58], a PIC formulation with broad lengths of dsRNAs (10–10,000 bp) was used for the experiments.

The JNK inhibitor SP600125, the ER stressor thapsigargin (Sigma) and the proteasome inhibitor MG-132 were used at concentration of 10 µM, 1 µM and 1 μM respectively [20], [34]. SP600125 was added to the cell medium 4h before PIC transfection and maintained during the whole period of PIC exposure.

### Coxsackievirus infection

The prototype strain of enterovirus (CVB5/Faulkner) was obtained from American Type Culture Collection (Manassas, VA). This virus was passaged in Green Monkey Kidney cells. The identity of the enterovirus preparations used was confirmed using a plaque neutralization assay with type-specific antisera [12].

Cells were infected with different multiplicity of infection (MOIs) of the virus preparation diluted in Hanks' Balanced Salt Solution (HBSS, Invitrogen). After adsorption for 1h at 37°C, the inoculum virus was removed, and the cells were washed twice with HBSS. Culture medium was then added to the plates and the virus was allowed to replicate for the indicated periods.

### Infection with adenoviral vectors

INS-1E cells and primary FACS-purified rat beta cells were infected for 2 h at different MOIs with an adenovirus encoding Luciferase (Ad-Luc), or with an adenovirus encoding rat Mcl-1 (Vector Biolabs, Philadelphia, PA, USA) [20]. After 48 h of recovery cells were exposed to intracellular PIC for 24h.

### Assessment of cell viability

Cells were incubated for 15 minutes with the DNA-binding dyes Propidium Iodide (PI, 5 µg/ml, Sigma) and Hoechst 33342 (HO, 5 µg/ml, Sigma). Subsequently, two independent observers determined the percentage of viable, apoptotic and necrotic cells. One of the observers was unaware of sample identity and the agreement between the results obtained was >90%. At least 500 cells were counted in each experimental condition. Results are presented as percentage of apoptosis, calculated as number of apoptotic cells/total number of cells x 100. This method is quantitative and has been validated for use in primary beta cells and INS-1E cells by systematic comparison with electron microscopy, caspase 3 activation and DNA laddering [33], [34], [57], [59], [60]. The apoptotic index was calculated against the percentage of apoptotic cells in the untreated condition as previously described [27]. In selected experiments, apoptosis was confirmed by analysis of activated (cleaved) caspase 3 and 9, cytoplasmic cytochrome *c* release and BAX translocation to the mitochondria (see below).

### Assessment of cytochrome *c* release

Cells were harvested in cold PBS, centrifuged (500 g for 2 min), resuspended with 50 µl lysis buffer (75 mM NaCl, 1 mM NaH_2_PO_4_, 8 mM Na_2_PO_4_, 250 mM sucrose, 21 µg/µl aprotinin, 1 mM PMSF and 0.8 µg/µl digitonin) and vortexed for 30 s. Following centrifugation at 20,000 g for 1 min the supernatant was retrieved as the cytoplasmic fraction. The remaining pellet was resuspended in 50 µl lysis buffer containing a higher digitonin concentration (8 µg/µl), centrifuged at 20,000 g for 1 min and the supernatant retrieved as the mitochondrial fraction. Equal amounts of proteins were then resolved by 14% SDS-PAGE gel [22]. In addition to anti-cytochrome *c* (BD Biosciences), the immunoblots were probed with antibodies against β-actin and apoptosis-inducing factor (AIF) (Cell Signaling, Danvers, USA) corresponding to a cytoplasmic and a mitochondrial control, respectively.

### mRNA extraction and real time PCR

mRNA was obtained using the Dynabeads mRNA DIRECT kit (Invitrogen Dynal, Oslo, Norway), and reverse transcribed to obtain the complementary DNA [33]. This material was analyzed by real time PCR reaction using SYBR Green fluorescence on a LightCycler instrument (Roche, Manheim, Germany) and correlated to a standard curve. Expression of the gene of interest was then corrected for the housekeeping gene glyceraldehyde-3-phosphate dehydrogenase (GAPDH). The values are normalized by the highest value of each experiment considered as 1. GAPDH was chosen as housekeeping gene because its expression is not modified by PIC treatment in insulin producing cells [33], [61]. Primers sequences are described in Supplemental Table S2.

### Western blot analysis

Cells were washed with cold PBS and lysed with either Laemmli buffer or Phospho lysis buffer [4]. Equal amounts of proteins were then resolved by 10–14% SDS-PAGE gel and transferred to a nitrocellulose membrane. Immunoblot analysis was performed with antibodies targeting Mcl-1 (Biovision, CA, USA), PKR, total eIF2α (Santa Cruz Biotechnology, CA, USA), phospho-JNK, cleaved caspases 3 and 9, phospho-eIF2α, phospho-PERK, total PERK, Bcl-XL, Bcl-2, Bim (Cell Signaling), enterovirus-specific polyclonal rabbit antiserum (1/600; KTL-510) [12] or α-tubulin (Sigma), used as housekeeping protein. Horseradish peroxidase-conjugated donkey anti-rabbit or anti-mouse IgG were used as secondary antibodies (Lucron Bioproducts, De Pinte; Belgium). Immunoreactive bands were revealed using a chemiluminescent substrate (Thermo Scientific) and detected by a LAS-3000 CCD camera (Fujifilm, Tokyo, Japan). The densitometry of the bands was evaluated using the Aida Analysis software (Raytest, Straubenhardt, Germany).

### Immunoprecipitation

For the immunoprecipitation, INS-1E cells were lysed on ice using immunoprecipitation buffer [31]. Total proteins were quantified and used as the starting material for immunoprecipitations. Equal amounts of proteins were incubated with rabbit anti-BIM antibody overnight at 4°C with gentle rocking. Antibody-protein complexes were retrieved with a 50% protein A-agarose slurry (Santa Cruz Biotechnology), washed with immunoprecipitation buffer, resuspended in SDS sample buffer and then boiled to separate antibody-protein complex from the protein A. Samples were subjected to 14% SDS-PAGE gel and immunoblotted with anti-α-tubulin (Sigma), anti-Mcl-1 (Biovision) or anti-BIM antibody (Cell Signaling).

### Immunofluorescence

Beta cells were plated on polylysine-coated glass culture slides (BD Biosciences). Cells were fixed for 15 min in 4% paraformaldehyde, washed with PBS and permeabilized for 5 min in Triton X-100 0.1%. Slides were then blocked using goat serum 5% and incubated overnight at 4°C in the presence of rabbit anti-Bax (1/1000; Santa Cruz Biotechnology) plus mouse anti-ATP Synthase β (mitochondrial marker) (1/2000; BD Biosciences) or an enterovirus-specific rabbit antiserum (1/600; KTL-510). Cells were washed next morning with PBS and incubated for 1 h with the appropriate Alexa fluor 488 or 555-conjugated antibodies (1/1000; Invitrogen). After, cells were stained with Hoechst, mounted and photographed using fluorescence microscopy (Axio Imager, Carl Zeiss, Zaventem, Belgium) [20].

### Statistical analysis

Data are presented as mean ± SEM. Comparisons were performed by two-tailed paired Student's *t*-test or by ANOVA followed by Student's *t* test with Bonferroni correction, as adequate. A P value < 0.05 was considered as statistically significant.

### Gene IDs

Numbers were taken from GenBank at Pubmed: myeloid cell leukemia sequence 1 (Mcl-1) - 60430; BCL2-like 11 (Bim) - 64547; B-cell lymphoma 2 (Bcl-2) - 24224; Bcl-2 extra-large (Bcl-XL) - 24888; dsRNA-dependent protein kinase (PKR) - 54287; p53 Up-regulated Modulator of Apoptosis (PUMA) - 317673; Death Protein-5 (DP5) - 117271; c-jun NH2-terminal kinase (JNK) - 116554; eukaryotic translation initiation factor 2A (eIF2α) - 502531; PRK-like endoplasmic reticulum kinase (PERK) - 29702.

## Supporting Information

Figure S1 Knockdown of Bcl-XL or Bcl-2 increase beta cell apoptosis. ***A*** *and* ***B.*** Western blot (top) and densitometry (bottom) of INS-1E cells transfected with dsRNA for different time points (n = 4–7, *P<0.05 vs. untreated). ***C*** *to* ***H.*** INS-1E cells were transfected with siControl or with specific siRNAs against Bcl-XL (siBcl-XL) or Bcl-2 (siBcl-2) and 48 h later exposed to internal dsRNA for 24 h. The knockdown of Bcl-XL (C) and Bcl-2 (D) was assessed by Western blot and quantified by densitometry. Results are normalized for the expression level of α-tubulin (n = 3, *P<0.01 vs. siControl, #P<0.05 vs. siControl + PIC). ***E*** *and* ***F.*** Apoptosis of INS-1E cells transfected with siControl, siBcl-XL or siBcl-2 and 48 later treated or not with dsRNA for 24 h was evaluated using HO/PI staining (n = 4, *P<0.01 vs. siControl, #P<0.01 vs. siControl + PIC). ***G*** *and* ***H.*** Cleaved caspases 9 and 3 and α-tubulin protein expression was evaluated by Western blot in the presence or not of Bcl-XL (G) or Bcl-2 (H) knockdown. Pictures are representative of 3 independent experiments. Data are mean ± SEM.
(TIF)

Figure S2 Confirmatory effects on beta cell viability with second and independent siRNAs against Bcl-XL or Bcl-2. ***A*** *and* ***B.*** INS-1E cells were transfected with siControl, siBcl-XL #2 (A) or siBcl-2 #2 (B) and 48 h after recovery exposed or not to internal dsRNA for 24 h. Cell viability was evaluated using HO/PI (n = 4–6, *P<0.01 vs. siControl, #P<0.01 vs. siControl + PIC). Data are mean ± SEM.
(TIF)

Figure S3 Mcl-1, but not Bcl-XL or Bcl-2, knockdown sensitizes INS-1E cells to dsRNA-induced apoptosis. ***A*** *to* ***C.*** Cells were transfected with siControl or with two different siRNAs against Mcl-1 (siMcl-1 and siMcl-1 #2), Bcl-XL (siBcl-XL and siBcl-XL #2) or Bcl-2 (siBcl-2 and siBcl-2 #2) as described in Methods. 48 h after recovery, the cells were treated or not with internal dsRNA for 24 h. Apoptosis was measured using HO/PI staining and the apoptotic index was calculated as described previously using siControl, siMcl-1 (siMcl-1 and siMcl-1 #2), siBcl-XL (siBcl-XL and siBcl-XL #2) or siBcl-2 (siBcl-2 and siBcl-2 #2) without dsRNA exposure as respective baseline [27] (n = 4–5, *P<0.05 vs. siControl, **P<0.01 vs. siControl). Data are mean ± SEM.
(TIF)

Figure S4 A second siRNA against Bim also prevents dsRNA-induced beta cell apoptosis. ***A*** *and* ***B.*** Bim was silenced by using a second independent siRNA (siBim #2) for 24 h. The knockdown of Bim was assessed by Western blot (A), and the cell viability (B) by the use of nuclear dies 24 h after PIC exposure (n = 3–6, *P<0.01 vs. siControl, #P<0.01 vs. siControl + PIC). The pictures shown are representative of 3 independent experiments. Data are mean ± SEM.
(TIF)

Figure S5 DP5 and PUMA are not mediators of beta cell apoptosis triggered by internal dsRNA. ***A*** *to* ***D.*** DP5 and PUMA were silenced by using specific siRNAs for 48 h. The knockdown was assessed by real time RT-PCR (A and C), and the cell viability (B and D) by using nuclear dies 24 h after PIC exposure (n = 3–4, *P<0.01 vs. siControl, #P<0.01 vs. siControl + PIC). Data are mean ± SEM.
(TIF)

Figure S6 Intracellular dsRNA and CVB5 induce interferon β expression. ***A*** *and* ***B.*** INS-1E cells were exposed to dsRNA (A) or CVB5 (B) for different time points and interferon β mRNA expression was analyzed by RT-PCR (n = 3, *P<0.01 vs. untreated). ***C.*** Interferon β mRNA expression after 24 h of PIC exposure in primary beta cells and INS-1E cells (n = 3–4).
(TIF)

Figure S7 The Mcl-1 degradation induced by dsRNA is not dependent of JNK activation. Analysis of JNK phosphorylation and Mcl-1 protein expression in INS-1E cells treated with dsRNA for different time points in the presence or absence of a JNK chemical inhibitor (SP600125) was done by Western blot (left) and quantified by densitometry (right). Mcl-1 expression was normalized to α-tubulin (n = 4, *P<0.01 vs. untreated). The pictures shown are representative of 4 independent experiments. Data are mean ± SEM.
(TIF)

Figure S8 Internal dsRNA does not induce PERK phosphorylation. ***A*** *and* ***B.*** Cells were transfected with dsRNA, as described in Methods, and harvested at different time points. The ER stressor thapsigargin 1 µM was used as a positive control. Phosphorylation of PERK and eIF2α was determined by Western blot (A) and quantified by densitometry (B) relatively to total PERK and total eIF2α respectively (n = 5, *P<0.01 vs. untreated). The pictures shown are representative of 5 independent experiments. Data are mean ± SEM.
(TIF)

Table S1 siRNAs used for silencing of selected genes.
(DOC)

Table S2 Primer sequences and their respective PCR fragment lengths.
(DOC)
