# Supplementary figures and images for: Exposure to the Viral By-Product dsRNA or Coxsackievirus B5 Triggers Pancreatic Beta Cell Apoptosis via a Bim / Mcl-1 Imbalance
Source: PLoS Pathog. 2011 Sep 22;7(9):e1002267. doi: 10.1371/journal.ppat.1002267 (PMC3178579; doi:10.1371/journal.ppat.1002267)

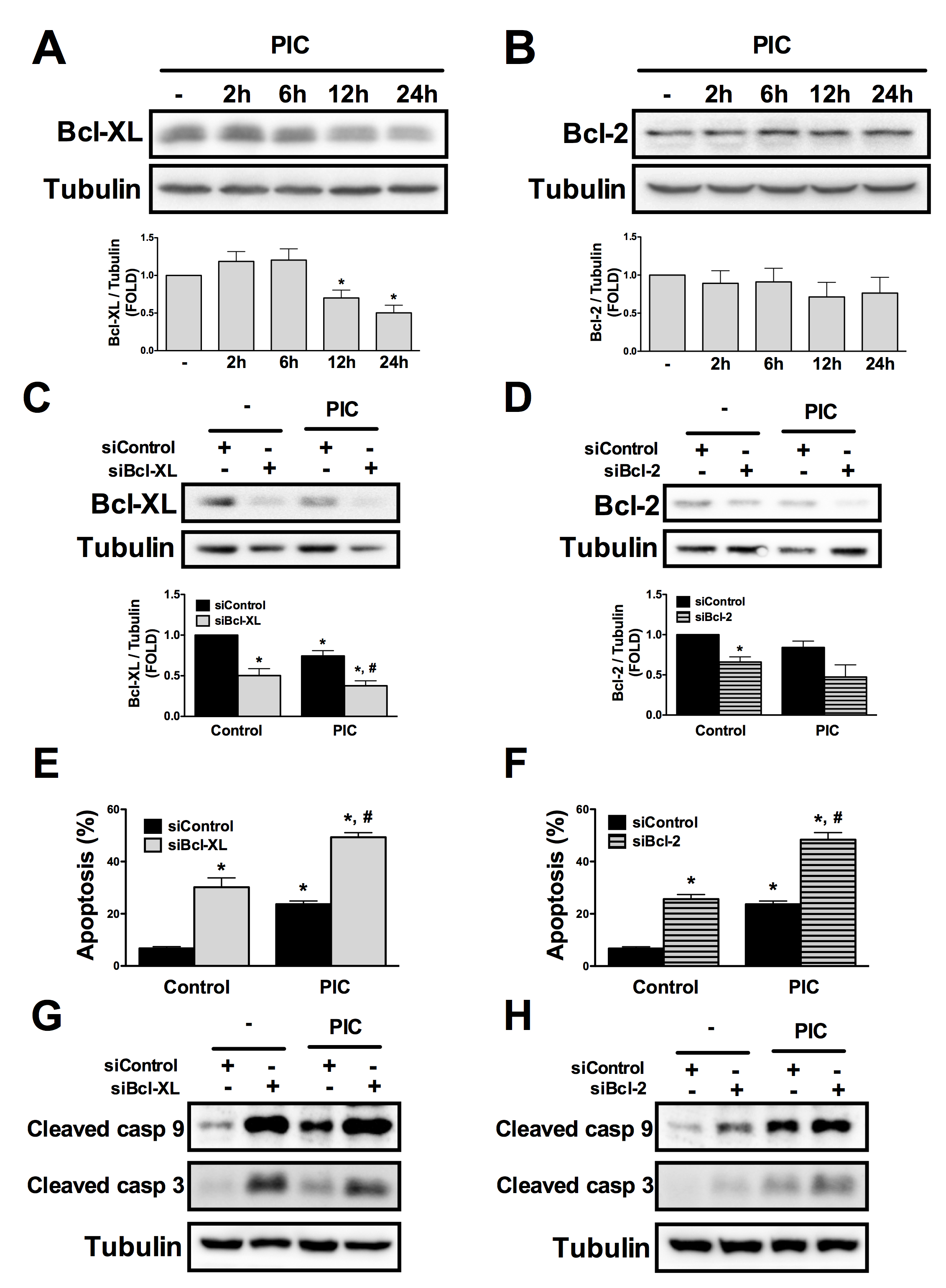

Supplement: Figure S1 — Knockdown of Bcl-XL or Bcl-2 increase beta cell apoptosis. A and B. Western blot (top) and densitometry (bottom) of INS-1E cells transfected with dsRNA for different time points (n = 4–7, *P<0.05 vs. untreated). C to H. INS-1E cells were transfected with siControl or with specific siRNAs against Bcl-XL (siBcl-XL) or Bcl-2 (siBcl-2) and 48 h later exposed to internal dsRNA for 24 h. The knockdown of Bcl-XL (C) and Bcl-2 (D) was assessed by Western blot and quantified by densitometry. Results are normalized for the expression level of α-tubulin (n = 3, *P<0.01 vs. siControl, #P<0.05 vs. siControl + PIC). E and F. Apoptosis of INS-1E cells transfected with siControl, siBcl-XL or siBcl-2 and 48 later treated or not with dsRNA for 24 h was evaluated using HO/PI staining (n = 4, *P<0.01 vs. siControl, #P<0.01 vs. siControl + PIC). G and H. Cleaved caspases 9 and 3 and α-tubulin protein expression was evaluated by Western blot in the presence or not of Bcl-XL (G) or Bcl-2 (H) knockdown. Pictures are representative of 3 independent experiments. Data are mean ± SEM. (TIF) [file ppat.1002267.s001.tif]

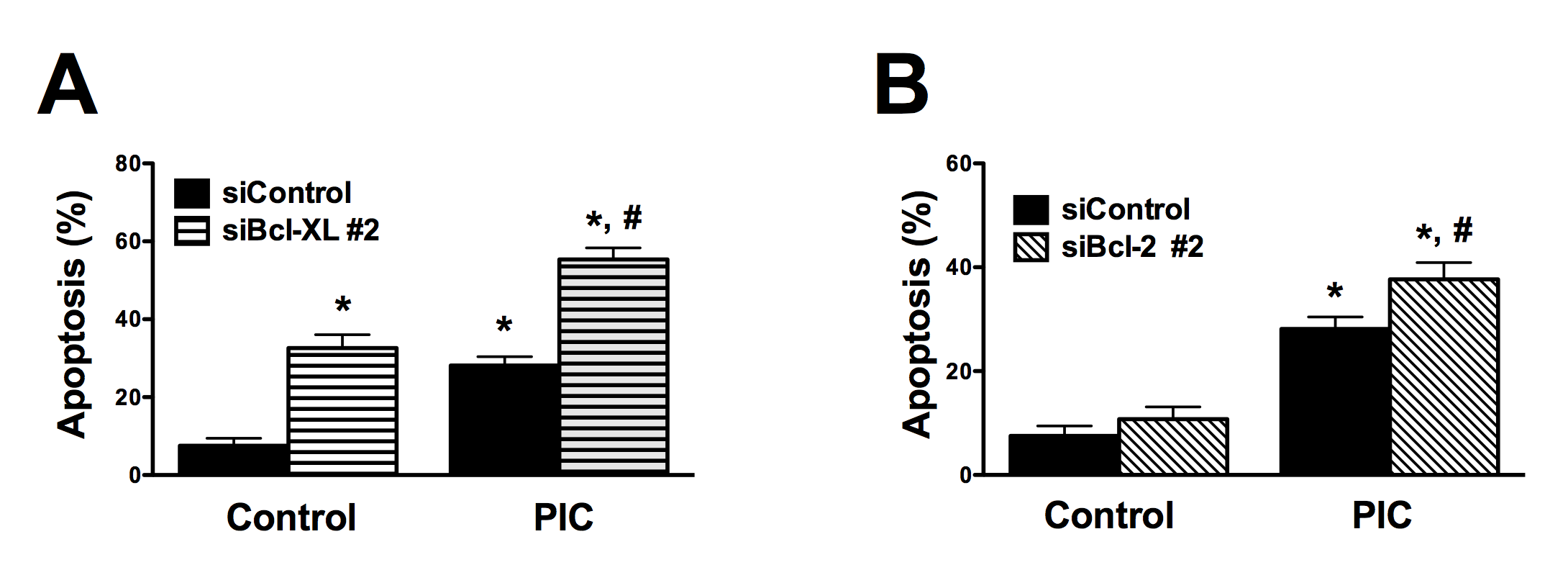

Supplement: Figure S2 — Confirmatory effects on beta cell viability with second and independent siRNAs against Bcl-XL or Bcl-2. A and B. INS-1E cells were transfected with siControl, siBcl-XL #2 (A) or siBcl-2 #2 (B) and 48 h after recovery exposed or not to internal dsRNA for 24 h. Cell viability was evaluated using HO/PI (n = 4–6, *P<0.01 vs. siControl, #P<0.01 vs. siControl + PIC). Data are mean ± SEM. (TIF) [file ppat.1002267.s002.tif]

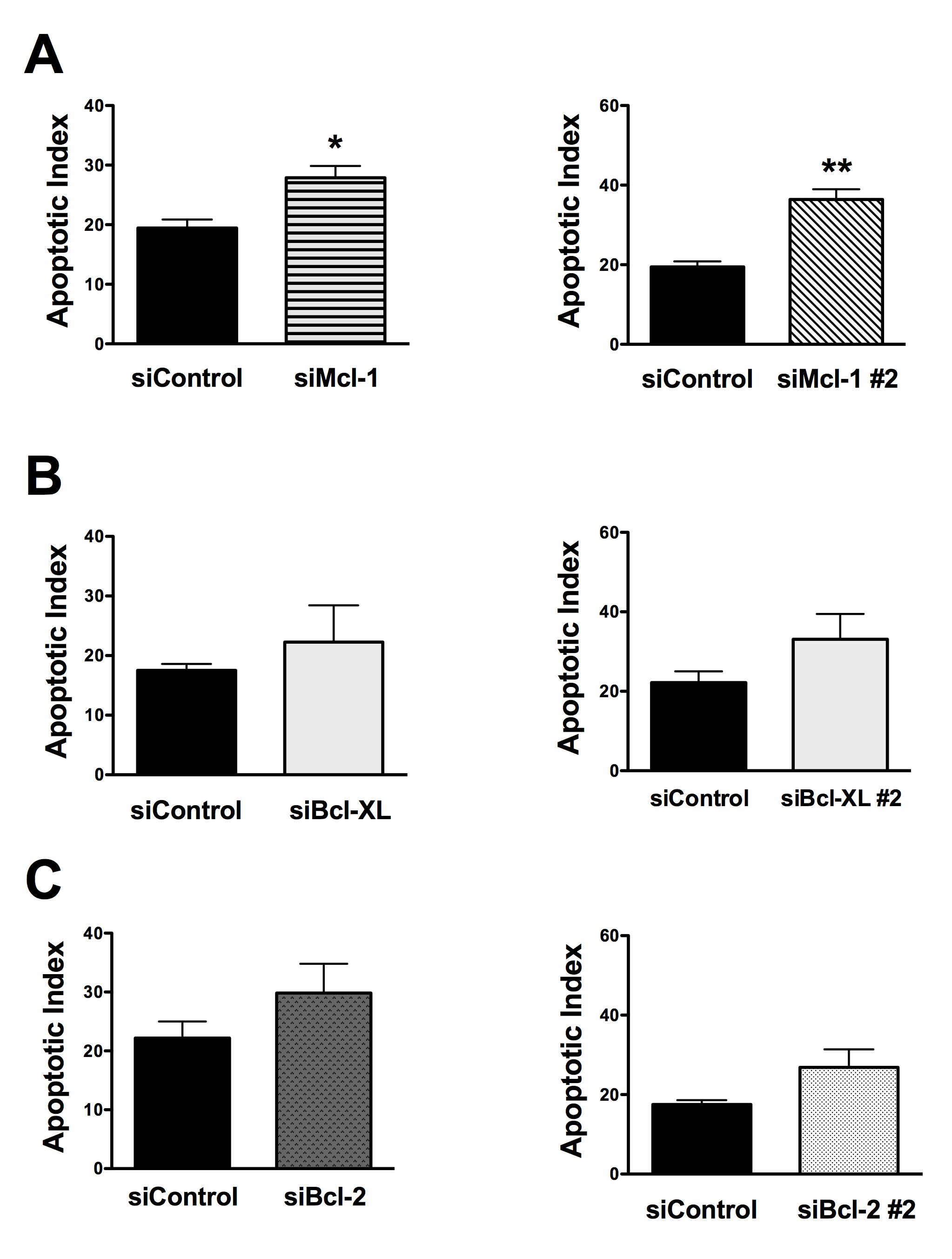

Supplement: Figure S3 — Mcl-1, but not Bcl-XL or Bcl-2, knockdown sensitizes INS-1E cells to dsRNA-induced apoptosis. A to C. Cells were transfected with siControl or with two different siRNAs against Mcl-1 (siMcl-1 and siMcl-1 #2), Bcl-XL (siBcl-XL and siBcl-XL #2) or Bcl-2 (siBcl-2 and siBcl-2 #2) as described in Methods. 48 h after recovery, the cells were treated or not with internal dsRNA for 24 h. Apoptosis was measured using HO/PI staining and the apoptotic index was calculated as described previously using siControl, siMcl-1 (siMcl-1 and siMcl-1 #2), siBcl-XL (siBcl-XL and siBcl-XL #2) or siBcl-2 (siBcl-2 and siBcl-2 #2) without dsRNA exposure as respective baseline [27] (n = 4–5, *P<0.05 vs. siControl, **P<0.01 vs. siControl). Data are mean ± SEM. (TIF) [file ppat.1002267.s003.tif]

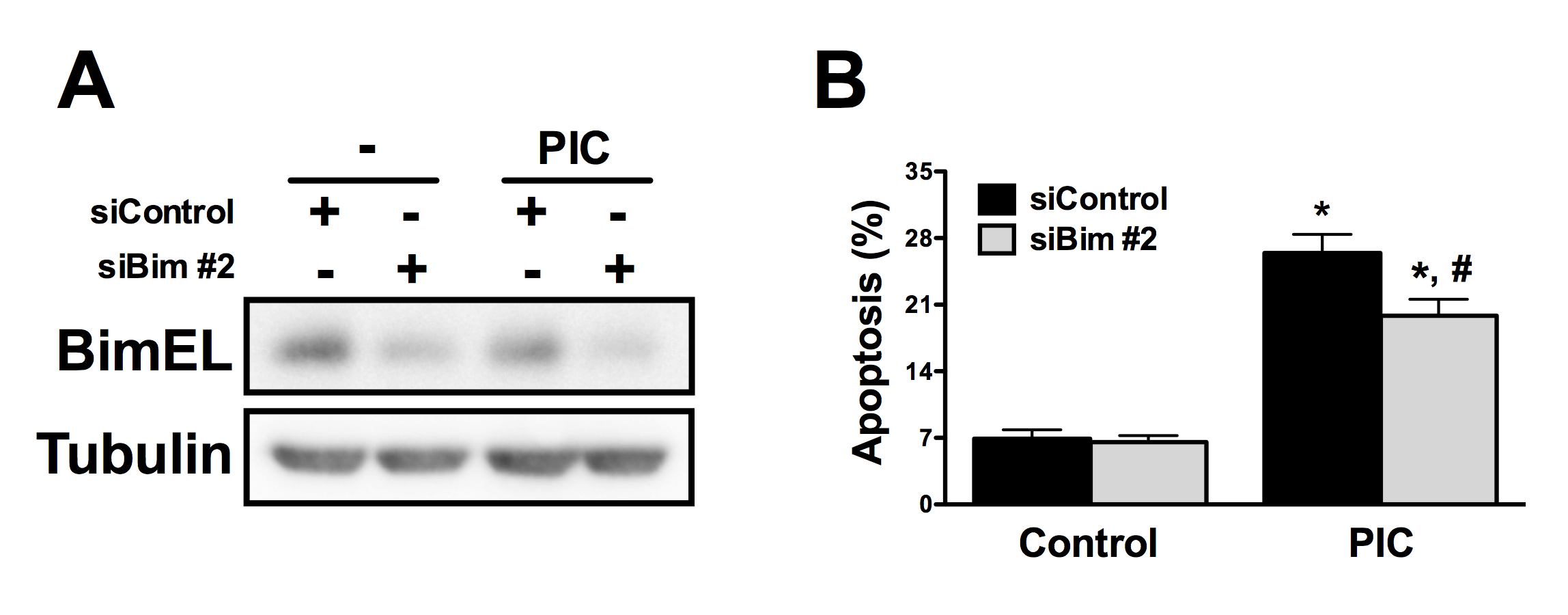

Supplement: Figure S4 — A second siRNA against Bim also prevents dsRNA-induced beta cell apoptosis. A and B. Bim was silenced by using a second independent siRNA (siBim #2) for 24 h. The knockdown of Bim was assessed by Western blot (A), and the cell viability (B) by the use of nuclear dies 24 h after PIC exposure (n = 3–6, *P<0.01 vs. siControl, #P<0.01 vs. siControl + PIC). The pictures shown are representative of 3 independent experiments. Data are mean ± SEM. (TIF) [file ppat.1002267.s004.tif]

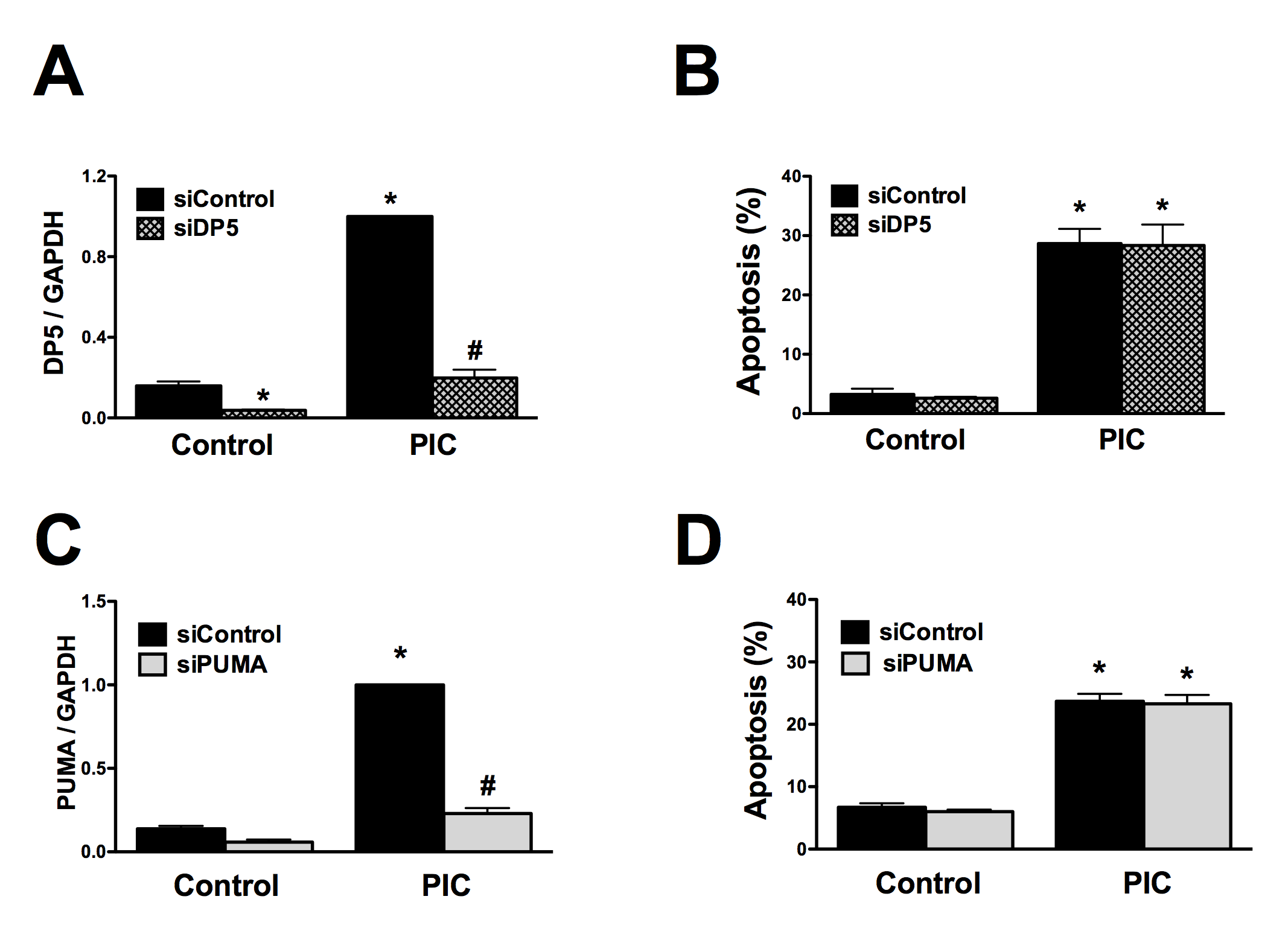

Supplement: Figure S5 — DP5 and PUMA are not mediators of beta cell apoptosis triggered by internal dsRNA. A to D. DP5 and PUMA were silenced by using specific siRNAs for 48 h. The knockdown was assessed by real time RT-PCR (A and C), and the cell viability (B and D) by using nuclear dies 24 h after PIC exposure (n = 3–4, *P<0.01 vs. siControl, #P<0.01 vs. siControl + PIC). Data are mean ± SEM. (TIF) [file ppat.1002267.s005.tif]

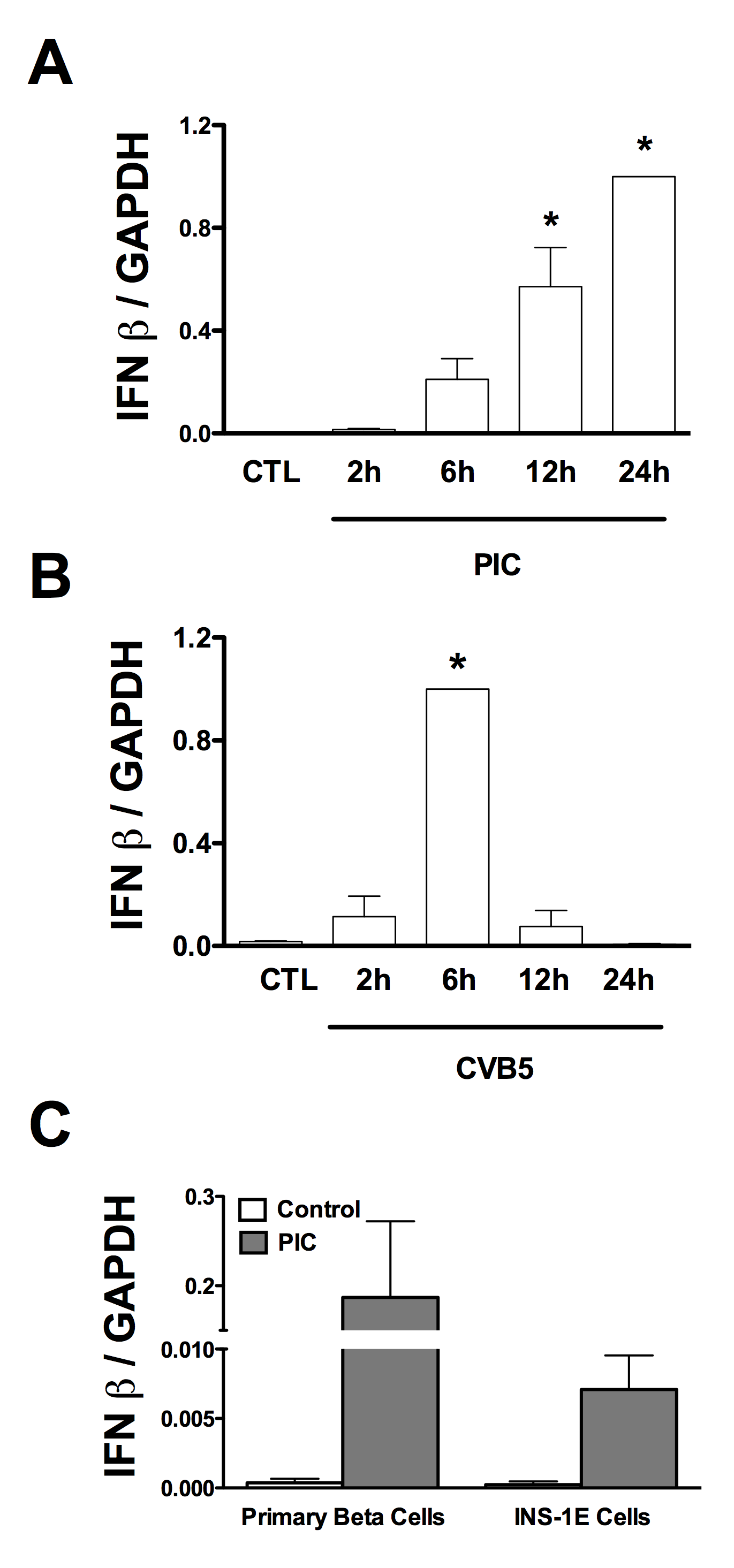

Supplement: Figure S6 — Intracellular dsRNA and CVB5 induce interferon β expression. A and B. INS-1E cells were exposed to dsRNA (A) or CVB5 (B) for different time points and interferon β mRNA expression was analyzed by RT-PCR (n = 3, *P<0.01 vs. untreated). C. Interferon β mRNA expression after 24 h of PIC exposure in primary beta cells and INS-1E cells (n = 3–4). (TIF) [file ppat.1002267.s006.tif]

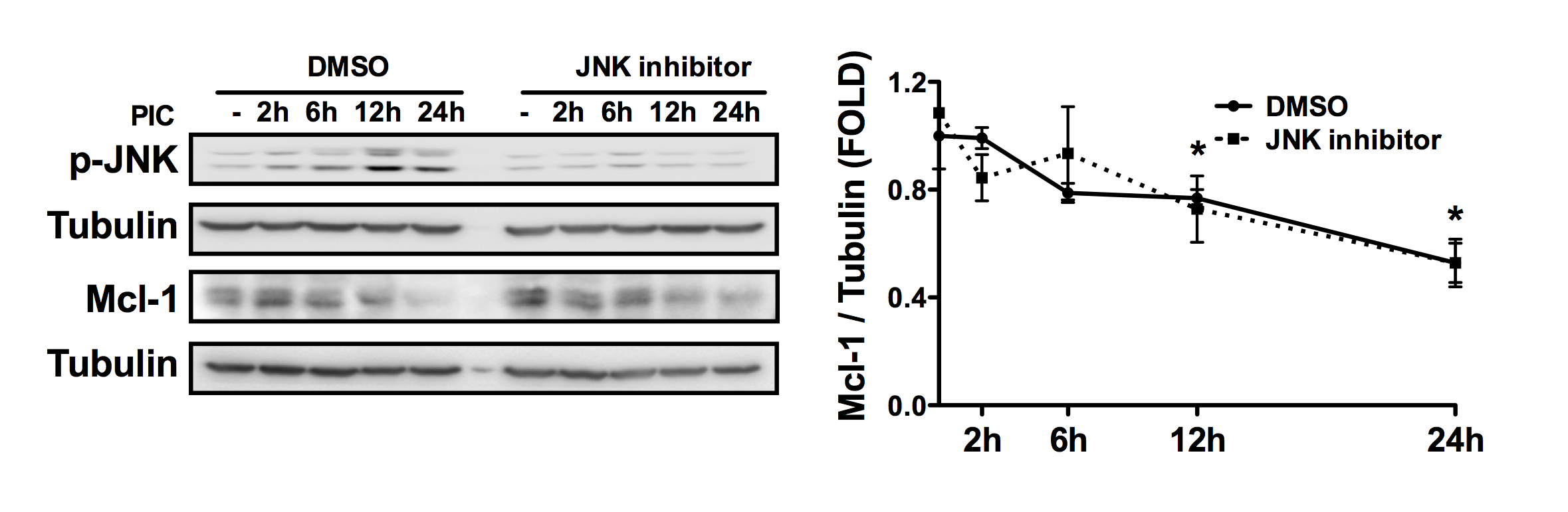

Supplement: Figure S7 — The Mcl-1 degradation induced by dsRNA is not dependent of JNK activation. Analysis of JNK phosphorylation and Mcl-1 protein expression in INS-1E cells treated with dsRNA for different time points in the presence or absence of a JNK chemical inhibitor (SP600125) was done by Western blot (left) and quantified by densitometry (right). Mcl-1 expression was normalized to α-tubulin (n = 4, *P<0.01 vs. untreated). The pictures shown are representative of 4 independent experiments. Data are mean ± SEM. (TIF) [file ppat.1002267.s007.tif]

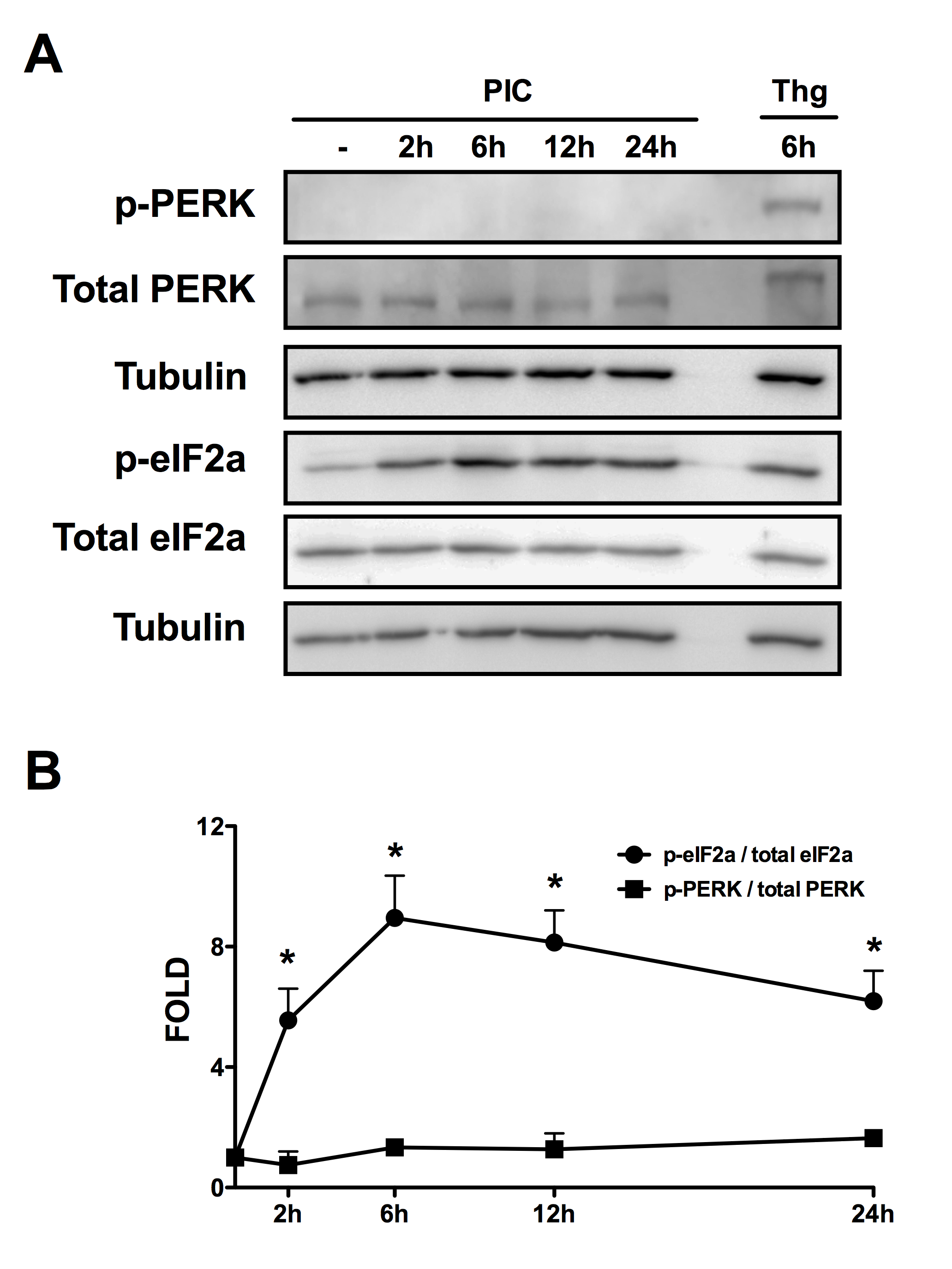

Supplement: Figure S8 — Internal dsRNA does not induce PERK phosphorylation. A and B. Cells were transfected with dsRNA, as described in Methods, and harvested at different time points. The ER stressor thapsigargin 1 µM was used as a positive control. Phosphorylation of PERK and eIF2α was determined by Western blot (A) and quantified by densitometry (B) relatively to total PERK and total eIF2α respectively (n = 5, *P<0.01 vs. untreated). The pictures shown are representative of 5 independent experiments. Data are mean ± SEM. (TIF) [file ppat.1002267.s008.tif]
