# Supplementary material for: Exposure to the Viral By-Product dsRNA or Coxsackievirus B5 Triggers Pancreatic Beta Cell Apoptosis via a Bim / Mcl-1 Imbalance
Source: PLoS Pathog. 2011 Sep 22;7(9):e1002267. doi: 10.1371/journal.ppat.1002267 (PMC3178579; doi:10.1371/journal.ppat.1002267)
Supplement: Table S1 — siRNAs used for silencing of selected genes. (DOC) [file ppat.1002267.s009.doc]

**Table S1.** siRNAs used for silencing of selected genes.

| **siRNA** | **Supplier** | **Sequence** |
| --- | --- | --- |
| Allstars Negative Control siRNA | Qiagen, Venlo, Netherlands |  |
| Rat Mcl-1 siRNA | App. Biosystems, Austin, USA | 5’- CGAGGACGAUGUUAAAUCU- 3’ |
| Rat Mcl-1 #2 SMARTpool® siRNA | Thermo Scientific, Chicago, USA | seq #1 5’- GUAAGGACGAAGCGGGACU- 3’ |
|  |  | seq #2 5’- GUAGAACAAAUCCGAGUUA- 3’ |
|  |  | seq #3 5’- GAAUUGUGGCUAACGAGAA- 3’ |
|  |  | seq #4 5’- GAUCAGUUCUAGUGUAUAU- 3’ |
| Rat Bim siRNA | Invitrogen, Carlsbad, USA | 5’- CGAGGAGGGCGUUUGCAAACGAUUA- 3’ |
| Rat Bim #2 siRNA | Invitrogen, Carlsbad, USA | 5’- GAGUUCAAUGAGACUUACACGAGGA- 3’ |
| Rat Bcl-XL siRNA | Invitrogen, Carlsbad, USA | 5’- AGAGAAAGUCAACCACCAGCUCCCG- 3’ |
| Rat Bcl-XL #2 siRNA | Invitrogen, Carlsbad, USA | 5’- GCGUAGACAAGGAGAUGCAGGUAUU- 3’ |
| Rat Bcl-2 siRNA | Invitrogen, Carlsbad, USA | 5’- GGAGAUCGUGAUGAAGUACAUCCAU- 3’ |
| Rat Bcl-2 #2 siRNA | Invitrogen, Carlsbad, USA | 5’- CAAGCCGGGAGAACAGGGUAUGAUA- 3’ |
| Rat DP5 siRNA | Invitrogen, Carlsbad, USA | 5’- UCACAGUUUCUUGGUGCUAAGUGUA- 3’ |
| Rat PUMA siRNA | Invitrogen, Carlsbad, USA | 5’- ACGAGCGGCGGAGACAAGAAGAGCA - 3’ |
| Rat PKR siRNA | App. Biosystems, Austin, USA | 5’- GGAAUUCUGUGAUAAAGGA- 3’ |
