# Supplementary material for: Exposure to the Viral By-Product dsRNA or Coxsackievirus B5 Triggers Pancreatic Beta Cell Apoptosis via a Bim / Mcl-1 Imbalance
Source: PLoS Pathog. 2011 Sep 22;7(9):e1002267. doi: 10.1371/journal.ppat.1002267 (PMC3178579; doi:10.1371/journal.ppat.1002267)
Supplement: Table S2 — Primer sequences and their respective PCR fragment lengths. (DOC) [file ppat.1002267.s010.doc]

**Table S2.** Primer sequences and their respective PCR fragment lengths.

| **Name** | **Forward** | **Reverse** | **lengths** |
| --- | --- | --- | --- |
| DP5 ST | 5’- CATGTCCTGTATGCCACCTG -3’ | 5’- GCTCAGACGTGGAGGTCTTC -3’ | 612 bp |
| DP5 RT | 5’- GCCGTGGTGTTACTTGGA -3’ | 5’- GATTGTGCCAGAGCTTCACA -3’ | 125 bp |
| GAPDH ST | 5’- ATGACTCTACCCACGGCAAG -3’ | 5’- TGTGAGGGAGATGCTCAGTG -3’ | 930 bp |
| GAPDH RT | 5’- AGTTCAACGGCACAGTCAAG -3’ | 5’- TACTCAGCACCAGCATCACC -3’ | 136 bp |
| Mcl-1 ST | 5’- AAACCTCCAGCCACCAACTA -3’ | 5’- CATCCTCCTTCACCCTCTTGTA -3’ | 456 bp |
| Mcl-1 RT | 5’- CCTCCAGCCACCAACTACAT -3’ | 5’- CCACTTTCTTTCTGCCGTGTTA -3’ | 97 bp |
| PUMA ST | 5’- TGGGTGCACTGATGGAGATA -3’ | 5’- AACCTATGCAATGGGATGGA -3’ | 497 bp |
| PUMA RT | 5’- AGTGCGCCTTCACTTTGG -3’ | 5’- CAGGAGGCTAGTGGTCAGGT -3’ | 110 bp |
| Interferon β ST | 5’-CTGCCCTCTCCATCGACTAC-3’ | 5’-TCCGAGCAGAAGTCTT-3’ | 453 bp |
| Interferon β RT | 5’-GCCTTTGCCATTCAAG-3’ | 5’-AGACAGAGCTTCTGGA-3’ | 131 bp |

ST: Standard curve; RT: Real time RT-PCR
